# Plasmalogens in Innate Immune Cells: From Arachidonate Signaling to Ferroptosis

**DOI:** 10.3390/biom14111461

**Published:** 2024-11-18

**Authors:** Jesús Balsinde, María A. Balboa

**Affiliations:** 1 Instituto de Biología y Geneética Molecular, Consejo Superior de Investigaciones Científicas Uva, 47003 Valladolid, Spain; 2 Centro de Investigación Biomédica en Red de Diabetes y Enfermedades Metabólicas Asociadas (CIBERDEM), Instituto de Salud Carlos III, 28029 Madrid, Spain

**Keywords:** plasmalogens, arachidonic acid, polyunsaturated fatty acids, inflammation, innate immunity, ferroptosis

## Abstract

Polyunsaturated fatty acids such as arachidonic acid are indispensable components of innate immune signaling. Plasmalogens are glycerophospholipids with a vinyl ether bond in the sn-1 position of the glycerol backbone instead of the more common sn-1 ester bond present in “classical” glycerophospholipids. This kind of phospholipid is particularly rich in polyunsaturated fatty acids, especially arachidonic acid. In addition to or independently of the role of plasmalogens as major providers of free arachidonic acid for eicosanoid synthesis, plasmalogens also perform a varied number of functions. Membrane plasmalogen levels may determine parameters of the plasma membrane, such as fluidity and the formation of microdomains that are necessary for efficient signal transduction leading to optimal phagocytosis by macrophages. Also, plasmalogens may be instrumental for the execution of ferroptosis. This is a nonapoptotic form of cell death that is associated with oxidative stress. This review discusses recent data suggesting that, beyond their involvement in the cellular metabolism of arachidonic acid, the cells maintain stable pools of plasmalogens rich in polyunsaturated fatty acids for executing specific responses.

## 1. Plasmalogens: Structure and Functions

**Figure 1:**
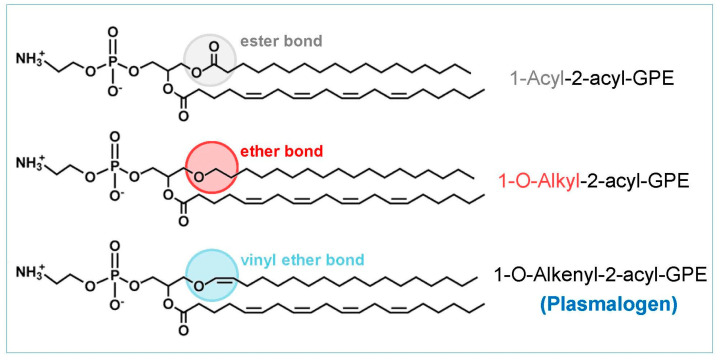
Structure of membrane glycerophospholipids. Most glycerophospholipids contain a fatty acid esterified at the sn-1 position of the glycerol backbone (1-acyl-2-acyl-). However, some instead feature a fatty acid alcohol at the sn-1 position, creating an ether bond (1-O-alkyl-2-acyl-). A subset of these contains in addition a double bound conjugated with the ether oxygen, called a vinyl ether (1-O-alkenyl-2-acyl-). These are the plasmalogens. GPE, glycerophosphoethanolamine.

Not all membrane glycerophospholipids share the same chemical structure. While most membrane glycerophospholipids contain two fatty acids, a minor fraction does not, because in the sn-1 position, there is a fatty alcohol. In addition, some possess a double bond conjugated to the oxygen of the ether bond; these are called plasmalogens (Figure 1). In mammalian cells, the plasmalogens typically have either choline or ethanolamine as the sn-3 headgroup. Choline plasmalogens are particularly abundant in heart and smooth muscle, whereas ethanolamine plasmalogens are the predominant form in the other organs. In innate immune cells such as monocytes and macrophages, ethanolamine plasmalogens are especially prevalent, while choline plasmalogen levels are generally low. Since this review focuses on innate immune cells, references to plasmalogens will, unless otherwise noted, specifically refer to ethanolamine plasmalogens.

Chemically, the ethanolamine plasmalogens are not phosphatidylethanolamines, as they derive from plasmenic acid (l-O-alk-l′-enyl-2-acyl-sn-glycero-3-phosphate), not phosphatidic acid (1,2-diacyl-sn-glycero-3-phosphate). Hence, ethanolamine plasmalogens can also be referred to as plasmenylethanolamines. The denomination “phosphatidylethanolamine plasmalogen”, although frequently found in the literature, is incorrect and should be avoided.

The plasmalogens serve a number of important roles in physiology and pathophysiology. For example, as they lack the sn-1 carbonyl, packing is easier, which increases membrane rigidity [1,2]. Ethanolamine plasmalogens are frequently found as components of specific membrane microdomains called lipid rafts. The relative plasmalogen content within these domains may affect key properties, such as fluidity, tendency to fusion, packing, thickness, and density, thereby influencing the biological behavior of membranes in transmembrane transport and signaling. Because of the presence of the vinyl ether bond, the plasmalogens are also suggested to act as endogenous scavengers of reactive oxygen species [1,2,3,4,5]. The recent discovery that the orphan gene TMEM189 encodes plasmanylethanolamine desaturase 1 (PEDS1)—the enzyme responsible for introducing the defining double bond in plasmalogens—marks a significant turning point in advancing our understanding of the biological role of plasmalogens, which is expected to increase rapidly [6,7,8,9].

Plasmalogen actions appear to be exerted specifically on discrete signaling routes [2]. In macrophages, plasmalogens do not influence major responses, such as the eicosanoid biosynthetic route or the mechanisms for membrane phospholipid remodeling with polyunsaturated fatty acids (PUFA) (see Section 3 below). However, they appear crucial for innate immune cells to exhibit enhanced responses to bacterial lipopolysaccharide and to effect phagocytosis [10,11,12]. The latter response seems to involve a direct effect of plasmalogens on the mitogen-activated protein kinase signaling cascade [2,12]. Moreover, recent data have highlighted the importance of plasmalogen biosynthesis in regulating inflammation and promoting its resolution in a sterile acute inflammation model in zebrafish [13].

Another distinctive feature of plasmalogens is that they are very enriched in PUFAs, particularly arachidonic acid (AA). This is especially true in innate immune cells. For example, in murine peritoneal macrophages, PUFAs represent more than 90% of the total fatty acid present in ethanolamine plasmalogens, with AA being the most abundant [10,11,12]. Such an abundance of AA in plasmalogens has traditionally been thought to reflect the important role that these phospholipids must play in AA mobilization responses and the subsequent production of bioactive eicosanoids.

It is widely accepted that the major phospholipase A_2_ enzyme effecting the receptor-stimulated AA mobilization response, namely, group IVA cytosolic phospholipase A_2_ (often abbreviated as cPLA_2_α), exhibits a clear preference for AA-containing phospholipid substrates [14,15]. Interestingly, recent careful in vitro analyses based on mass spectrometry determinations have indicated that cPLA_2_α indeed manifests a certain degree of selectivity for AA-containing plasmalogen forms over conventional diacylphospholipids [16]. While this unanticipated finding may provide a molecular logic for the presence of high levels of AA in plasmalogens, it is important to note that conditions of in vitro assays may not necessarily correspond with the in vivo conditions, where compartmentalization of substrates and products and the presence of competing enzymes may alter the degree of hydrolysis of the different phospholipid substrates. Mass-spectrometry-based lipidomic analyses in activated cells do not show obvious differences between the hydrolysis of plasmalogens and diacylphospholipids by cPLA_2_α [17,18,19], suggesting that the substrate specificity of cPLA_2_α in cells may also be limited by its subcellular localization and, hence, the phospholipid composition of that compartment. In this regard, another recent lipidomic study in macrophages highlighted the importance of the selective hydrolysis of AA-containing inositol phospholipids by cPLA_2_α [20].

## 2. Direct Transfer of AA Among Phospholipids: Coenzyme A-Independent Transacylase

The plasmalogen enrichment with AA is long known to be due to the existence of a constitutive transfer of AA moieties from choline glycerophospholipids (PC) to ethanolamine glycerophospholipids (PE) that operates after but independently of the well-established Lands cycle of phospholipid fatty acid recycling. While the latter is CoA-dependent and may use any fatty acid, the former is strikingly CoA-independent and utilizes PUFA [21,22,23]. The enzyme carrying out this reaction is called CoA-independent transacylase (CoA-IT; EC 2.3.1.147), and catalyzes the CoA-independent transfer of AA and other PUFA primarily from diacyl-PC to several lysophospholipid molecular species. Strikingly, it manifests a strong affinity for lysophospholipid acceptors that possess an ether bond in the sn-1 position of the glycerol backbone, and the preference is remarkable when the acceptor is an ethanolamine lysoplasmalogen. This not only explains the enrichment of plasmalogens with AA but also the finding that plasmalogens generally contain more AA than their diacyl counterparts [21,22,23].

Despite the CoA-IT enzyme activity having been identified more than 40 years ago, the CoA-IT gene sequence remained persistently elusive. The enzyme activity has been extensively characterized in vitro and, in cells, it has been readily followed by determining the transfer of radiolabeled AA from PC to PE [24].

Notably, last year, Reed et al. reported that the TMEM164 gene product, which had earlier been defined as an important regulator of ferroptosis [25], catalyzes the entry of AA into the ethanolamine plasmalogen fraction via a direct transacylation reaction that uses AA-containing PC as a donor [26]. The deletion of this gene provides protection from ferroptotic cell death to a number of ferroptosis-sensitive cancer cell lines [26]. Thus, in addition to providing evidence for the regulatory role of AA-containing plasmalogens in ferroptosis, these findings are significant, because they establish that TMEM164 is a CoA-IT that moves AA among phospholipids—the first one to be identified at the gene level. This groundbreaking discovery paves the way for a more detailed understanding of the regulatory mechanisms of this key enzyme in cellular PUFA metabolism and signaling.

Whether, in addition to TMEM164, the cells express other enzymes with CoA-IT activity remains to be established. It appears likely that this will be the case, given the relatively high number of phospholipid AA remodeling enzymes (i.e., CoA-dependent acyltransferases and phospholipase A_2_s) that most cells express [27,28]. Of note, TMEM164-deficient cells showed the expected decreases in plasmalogen-bound AA, but this was not accompanied by an increase in AA in diacyl-PE, as it occurs in plasmalogen-deficient cells [24] (see Section 3 below). This raises the possibility that the TMEM164-deficient cells may be marginally deficient in AA, even though they show elevated levels of AA in diacyl-PC [26]. These are interesting observations, because the early characterizations of the CoA-IT reaction in macrophage extracts did establish that, although lysoplasmalogen (1-alkenyl-2-lyso-PE) is a preferred acceptor, lysoPE (1-acyl-2-lyso-PE) can also be used by CoA-IT to a significant extent [23,29].

## 3. Role of PE Molecular Species in AA Release and Metabolism

Given the high enrichment of plasmalogens in AA, it would seem logical to expect that these phospholipids constitute a major source of free fatty acid for lipid mediator production. Several decades ago, Zoeller and colleagues established two plasmalogen-deficient macrophage cell lines [30]. Recent mass spectrometry-based lipidomic analyses of these cells confirmed the near-complete absence of plasmalogens, while also revealing a compensatory increase in AA levels in diacyl-PE, in a manner that the total cellular AA content and distribution are preserved among all phospholipid classes [24]. In turn, this also indicates that plasmalogen deficiency does not hinder CoA-IT-mediated AA remodeling from PC to PE [24]. The analysis of stimulus-induced AA mobilization in these plasmalogen-deficient cells using either radioactivity measurements [31] or actual mass levels [24], produced the unexpected finding that the plasmalogen-deficient cells mobilize free AA and synthesize eicosanoids at levels comparable to those of parent cells with normal plasmalogen levels. These data emphasize that the distribution of AA among the multiple phospholipid species present in innate immune cells will depend primarily on the polar headgroup (i.e., PC versus PE versus PI) rather than on the chemical nature of the bond at the sn-1 position (i.e., acyl versus alkyl versus alkenylacyl species). In summary, if a full AA response can be achieved even in the absence of plasmalogens, and the phospholipase A_2_s involved utilize ester phospholipids equally well, without any effect on the amount of released AA [15,31], it suggests that the plasmalogen content of the macrophages does not influence their AA mobilization responses.

Importantly as well, recent studies utilizing double-labeling with radioactive AA of the various cellular pools under conditions that allow distinction of the fatty acid present in each major phospholipid class revealed that macrophages engaged in the phagocytosis of yeast zymosan particles produce prostaglandins that derive primarily from PC and, to a lesser extent, from PI. The contribution of PE molecular species appears to be minor [32]. These results support the notion that specific phospholipid AA pools are linked to the production of select eicosanoids during cell activation. Hence, the nature and quantity of eicosanoids produced under activation conditions may ultimately depend on compartmentalization, i.e., the composition and subcellular localization of the phospholipid pool where the AA-hydrolyzing phospholipase A_2_ acts.

The unexpected behavior of the PE class of phospholipids regarding AA dynamics does not end here. Utilizing mass spectrometry analyses, it was initially found in murine macrophages [18], later confirmed in primary monocytes [33], and again in murine macrophages [10,17] that the mass amount of AA in the PE fraction changes little after activation of the cells with yeast-derived zymosan. These studies align well with earlier work using mast cells labeled with radioactive AA and exposed to antigen or ionophore A23187 [34], suggesting that these events occur not only in phagocytic cells but also in other innate immune cells. The finding that the AA content in PE shows minimal variation after cell activation does not mean in any manner that PE is not a source of releasable AA during innate immune activation; indeed, phospholipase A_2_ enzymes, particularly cPLA_2_α, accept AA-containing PE as a substrate quite readily [16,19]. Rather, it comes to emphasize the importance of CoA-IT-driven reactions in maintaining AA levels in PE.

While the direct acylation of lysoPC and lysoPI with AA by CoA-dependent acyltransferases is generally accepted as the main route for AA incorporation into PC and PI, the major route for AA incorporation into PE occurs via CoA-IT-mediated transacylation reactions [21,22,23]. The AA that is removed from PE by cPLA_2_α during macrophage activation is rapidly replenished by CoA-IT at the expense of, primarily, diacyl-PC, thus giving a misleading impression that PE does not contribute to overall AA release or does so to a very modest level. The actual contribution of PE to AA release can be easily uncovered when experiments are carried out in the presence of CoA-IT inhibitors that block the incorporation of AA into PE [17]. In the later study [17], the use of CoA-IT inhibitors also resulted in a decrease in the production of cyclooxygenase products. Similar results were also shown in work with human neutrophils, where both prostaglandin E_2_ and leukotriene C_4_ production were decreased in the presence of CoA-IT inhibitors [35]. Thus, while some studies indicate that PC is the primary source of AA for synthesizing certain eicosanoids [32,36,37], others suggest that it is not the only source of AA for eicosanoid biosynthesis [17,35].

**Figure 2:**
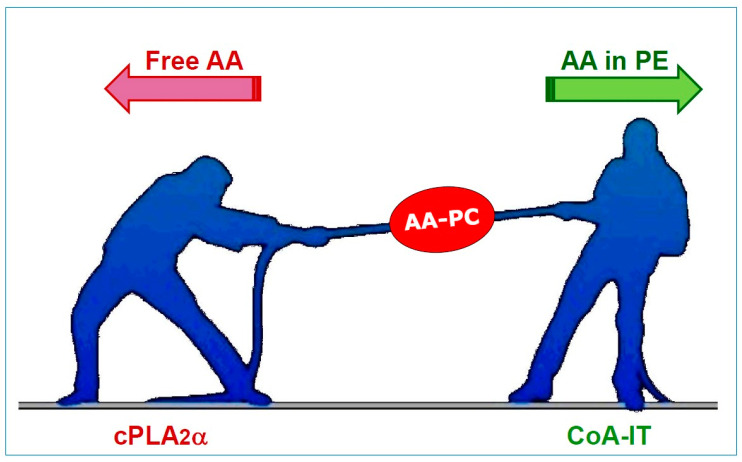
Dual role of AA-containing PC (AA-PC) in lipid signaling. The levels of AA within the PC class of phospholipids are influenced by two opposing reactions, resembling a tug-of-war. On the one hand, cytosolic phospholipase A_2_α (cPLA_2_α) acts on AA-PC to release free AA. On the other hand, coenzyme-A-independent transacylase (CoA-IT) transfers AA directly from PC to PE. By channeling AA from PC to PE, CoA-IT may effectively reduce the availability of substrate for cPLA_2_α, thereby limiting eicosanoid formation in activated macrophages.

On the other hand, the contribution of PC to overall AA release could be overestimated, because much of the AA lost from this species is not released as a free fatty acid but transferred to PE. Similar to a tug-of-war, the strong decrease in PC-bound AA that activated cells experience is due to both the actions of cPLA_2_α, which promotes free fatty acid formation, and CoA-IT, which moves the fatty acid towards PE (Figure 2). The relative contribution of the two reactions that control AA levels in PC during cell activation may represent an important regulatory point of the eicosanoid cascade, because the amount of PC-bound AA available for eicosanoid synthesis via cPLA_2_α will depend on how much AA is transferred to PE. Ultimately, while PC is the primary phospholipid class to lose AA upon cellular activation, this loss is strikingly influenced by the intermediate involvement of PE.

As a corollary of all the above, the finding that the levels of AA in PE species change little as a consequence of macrophage activation raises the intriguing possibility that the striking enrichment of PE with AA may not be primarily related to regulatory aspects of AA homeostasis and eicosanoid metabolism. Then, what would be the purpose for macrophages storing such a large amount of AA in PE, particularly in the plasmalogen fraction? Ferroptosis may provide clues to answer this question.

## 4. PE Molecular Species and Ferroptosis

**Figure 3:**
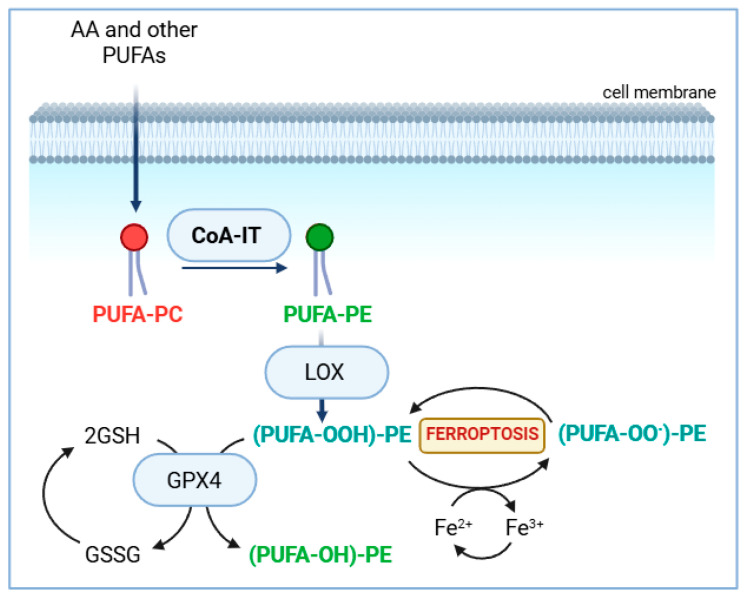
Ferroptosis at a glance. This form of cell death, driven by iron, results from the accumulation of phospholipid peroxides, particularly those derived from PUFA-containing PE. These include (PUFA-OOH)PE, a hydroperoxide of PUFA-containing PE, and (PUFA-OO•)PE, a peroxyl radical of PUFA-containing PE. The significance of the CoA-IT pathway in enriching PE with AA derived from PC is also illustrated. For further details please refer to text. GSH, glutathione; GSSG, oxidized glutathione; LOX, lipoxygenase.

Ferroptosis is a nonapoptotic form of cell death that, as its name implies, depends on iron. Unlike other forms of cell death, ferroptosis does not use a specific protein effector, like a pore-forming protein or a receptor. Rather, lipid peroxidation and the resultant membrane damage is the cause [38,39]. Under normal conditions, cells possess several mechanisms to control lipid peroxidation, which may occur either enzymatically, via lipoxygenases and cyclooxygenases, or spontaneously. A major defensive effector is glutathione peroxidase 4 (GPX4) [40]. When this enzyme fails, phospholipid hydroperoxides accumulate and, in the presence of iron, peroxyl radicals are generated via Fenton-like reactions, which help propagate a radical chain reaction [38,39]. The accumulation of hydroperoxide- and peroxyl-radical-containing phospholipids in membranes results in the irreversible damage of the integrity and stability of these membranes (Figure 3). A major difference between ferroptosis and apoptosis is that the former does not lead to cell shrinkage and plasma membrane blebbing [41,42].

In addition to GPX4, cells employ several other mechanisms to counteract lipoperoxide accumulation, including regulating intracellular Fe^2+^ and glutathione levels. However, one mechanism stands out in the context of this work: the overall cellular PUFA content. The more enriched a cell is in PUFA, the more susceptible it becomes to ferroptotic cell death [43,44,45]. Consequently, the pathways responsible for PUFA entry and redistribution into cellular phospholipids play a critical role in controlling cell sensitivity to ferroptosis. Specifically, the direct incorporation of PUFA into PC, primarily mediated by ACSL4 and LPCAT3 [27,28], along with the subsequent transfer of AA to PE, recently identified as being mediated by TMEM164 [26], emerge as promising targets for the pharmacological manipulation of ferroptosis.

While ferroptosis has been primarily studied in cancer cells, recent research has shown that innate immune cells are also highly susceptible to ferroptosis under the right conditions [45,46]. Innate immune cells, especially the macrophages, are very rich in PUFA. For instance, in mouse peritoneal macrophages, AA alone makes up 20–25% of the total fatty acids present in membrane phospholipids. The major reservoir of AA in these cells is PE, in particular the plasmalogen fraction, which accounts for approx. 40% of the total AA in these cells. Besides AA, macrophages contain significant amounts of AdA and DHA, both of which are also potent inducers of ferroptosis when peroxidized. Notably, AdA and DHA, like AA, also accumulate in the plasmalogen fraction of macrophages. The enrichment of PE molecular species, including plasmalogens, with AA and other PUFAs appears to play a critical role in the onset of ferroptosis. Repeated evidence has indicated that, of all possible oxidative processes in membrane phospholipids, the peroxidation of PE species—particularly those derived from plasmalogen-bound AA and AdA—serves a crucial role in driving ferroptosis under certain conditions [47,48,49,50,51,52,53]. It has been noted that, during ferroptosis, the ratio of oxidized PE species to oxidized non-PE species is higher than the ratio of total PE species to total non-PE species typically present in the inner plasma membrane of mammalian cells [43], suggesting that the oxidative process does not occur randomly. Moreover, PE-binding protein 1 (PEBP1) has been identified to interact specifically with PE species and 15-lipoxygenase, resulting in the selective accumulation of oxidized PE and the onset of ferroptosis [54,55,56].

## 5. Conclusions

In all, it may seem that AA accumulates so significantly in PE species, especially the plasmalogens, to provide the cells with a powerful and efficient means to die gracefully. Clearly further research in this area should yield valuable insights into the role and regulation of plasmalogen enrichment with PUFA and its pivotal role in cell function.

## Data Availability

Not applicable.

## Abbreviations

AA: arachidonic acid (*cis*-5,8,11,14-eicosatetraenoic acid, 20:4n–6); AdA, adrenic acid (*cis*-7,10,13,16-docosatetraenoic acid, 22:4n–6); DHA, docosahexaenoic acid (*cis*-4,7,10,13,16,19-docosahexaenoic acid, 22:6n–3); CoA-IT, coenzyme-A-independent transacylase; GPX4, glutathione peroxidase 4; PC, choline-containing glycerophospholipids; PE, ethanolamine-containing glycerophospholipids; PI, phosphatidylinositol; PUFA, polyunsaturated fatty acid.
